# Critical Transitions in Ecosystems and Society. The Contribution of Sociological Systems Theory to the Analysis of Socio-Environmental Transformations

**DOI:** 10.3389/fsoc.2021.763453

**Published:** 2022-01-24

**Authors:** Aldo Mascareño

**Affiliations:** ^1^ Centro de Estudios Públicos, Santiago, Chile; ^2^ Escuela de Gobierno, Universidad Adolfo Ibáñez, Santiago, Chile

**Keywords:** complexity, critical transitions, social-ecological systems, lock-in mechanism, systems theory, communication, self-descriptions, regime shifts

## Abstract

The theory of critical transitions and the theory of self-referential social systems are two well-established theories in the ecosystem and sociological research respectively. A dialogue between them may offer new insights on the complex articulation of the nature and society nexus in socio-environmental transformations. By means of the conceptual reconstruction of both theories and drawing on relevant literature of social-ecological research, in this article, I argue that systems theory can contribute to the theory of critical transitions with a robust concept of communication that accounts for the relevance of semantics and social structures, the production of communicative locks, and the identification of early warning signals of social-ecological transitions in communication. On the other hand, the theory of critical transitions provides systems theory with both a refined concept of crisis as critical transition and the technical tools for empirical research. The article concludes that the dialogue between the science of ecosystems and the science of society is not an intellectual exercise but a form of increasing the correspondence between social-ecological transitions and our explanations and interventions in this domain.

## 1 Introduction

One of the main problems in the sociological analysis of natural events consists in observing nature as an entity external to society; and one of the main problems of scientific research on natural dynamics consists in excluding or underrating the human factor from the equation. This was a central theoretical assumption inspiring Ulrich Beck’s *Risikogesellschaft* in the mid-1980s–the tipping point in contemporary sociology for considering the complex integration between nature and society. While the opposition between nature and society was a construction of the 19th century with the purpose of dominating and, at the same time, ignoring nature, much of the discussion on natural events and processes in the 20th century developed without humans, without the question on the social significance of naturals transformations (Beck, 1986). Several ecological crises in the 1970s affecting urban settlements, as well as critical failures in major technological systems, particularly regarding nuclear power stations such as Five Mile Island and Chernobyl, announced the era of complex risks (Luhmann, 2003) and normal accidents (Perrow, 1984). The proposal of Beck’s theory of reflexive modernization was to consider nature as a manufactured entity, where the manufacture and the possibility of total self-destruction was at expense of human beings (Beck, 1986; Beck et al., 1994).

Sharing a similar concern, other sociological theories began to be developed in the 1980s. On the one hand, actor-network theory (ANT) and science and technology studies (STS) preferred the network metaphor to construct their conceptual architectures (Callon et al., 1986), as well as the methodological emphasis on epistemological and ontological concerns (Callon, 1984; Latour, 1999; Law, 1999, Law, 2004, Latour, 2013). This connected ANT and STS to the post-structuralist turn in social sciences but made interdisciplinary dialogue with the empirical environmental sciences more difficult, since they themselves became an object of the *semiotics of materiality*. On the other hand, social systems theory adopted the distinction between system and environment and the concept of complexity as a form of reconstructing the conditions of autonomy and interdependence between different self-produced entities, namely, machines, organisms, social systems, and psychic systems (Luhmann, 1995). While Niklas Luhmann’s emphasis on the emergence of systems from a contingent world by means of self-referential operations placed the theory at the center of the post-structuralist and post-foundational turn (Moeller, 2012), the concept of complexity as well as the systematic theorization of categories coming from biology and complexity sciences -such as autopoiesis, structural coupling, operational closure, bifurcation, order from chaos, stability through instability and evolution− made the theory much more open to a fruitful dialogue with environmental sciences than ANT and STS were.

If approaching nature has been difficult for sociology from its very beginning, approaching society from natural sciences has not been always successful either. The same Cartesian distinction between society and nature that affected the conception of nature in social sciences (Aldeia and Alves, 2019) had also consequences for natural sciences. Rather, the ontological divide nature/society was reflected in Wilhelm Dilthey’s classical division between Natur-und Geisteswissenschaften (Dilthey, 1989), both sciences with radically different methods, empirical in the first case and interpretative in the second one. To that extent, exercises of crossing the line from natural into social sciences have always been more or less radically contested and problematic -e.g., social Darwinism and Wilson’s sociobiology (Hofstadter, 1992; Baxter, 2007)– and the integration of methods rejected almost ideologically–e.g., the Positivismusstreit between critical rationalism and critical theory (Adorno et al., 1972).

In the last two decades, however, things began to look more promising in social-ecological research for cross-fertilization between approaches coming from natural and social sciences. The keyword here is complexity. The convergence of ecological research, network science, graph theory, and the increased computational capacity for big data processing has enabled the empirical identification of dynamics and patterns that transcend the classical borders between nature, technology, and society, thereby allowing a proper mapping and understanding of the complex interrelationships between those domains. Theories such as panarchy (Gunderson and Holling, 2002), resilience theory (Berkes et al., 2003; Folke, 2006; Folke et al., 2016), and the theory of critical transitions (Scheffer, 2009) have shown that complexity is not a hypothesis but a real dynamic web including objects, events, and meanings that historically and topologically connect with each other at different levels, intensities, and periodicities, sometimes cyclically, sometimes suddenly and explosively. This presents a new scenario for attempts of capturing specific developments in sociological theory that may contribute to social-ecological research.

In this article, I focus on the particular intersection between the theory of critical transitions and social systems theory. I certainly do not exclude other possible cross-fertilizations, yet it seems to me that the emphasis on the connectivity of communicative events of social systems theory can contribute to assessing the performative effects of communication on social-ecological transitions. On the other hand, the theory of critical transitions provides social systems theory with a technical approach to systemic crises that is missing in systems analysis.

Although well-established, the theory of critical transitions can still benefit from specialized sociological knowledge and research for supplementing the view on social-ecological transitions. Consequently, I argue that Niklas Luhmann’s theory of social systems is best suited for contributing to a sociologically well-informed theory of critical transitions in socio-environmental research. Its emphasis on meaning (Sinn) and communication as basic concepts defining the social may offer crucial contributions at least at three levels: 1) social systems theory presents a way to observe and understand how the social description of environmental events have performative effects that might otherwise go unexplored when analyzing social-ecological transitions and transformations; 2) the self-produced character of social communication is prone to produce communicative locks that eventually trigger critical transition processes; social systems theory offers both an explanation to these communicative locks and conceptual tokens for their empirical identification; and 3) the sensibility of social communication to minimal environmental changes (particularly in local communities) may offer clear access points to multiple early warning signals anticipating critical transitions. Besides, technical indicators such as critical slowing down and flickering (Scheffer et al., 2015) have communicative correlates that can be observed through social systems analysis. Drawing on relevant literature of social-ecological research, I argue that social systems theory offers a complex view on social complexity with positive consequences for the analyses of critical transitions and transformations in socio-environmental settings as well as for the correspondence between these phenomena and our explanations.

To unfold this argument, I begin with a reconstruction of the theory of critical transitions in which I give particular attention to the system dynamics of social-ecological transitions, as well as to the mechanisms of behavioral lock-in and the role of early warning signals in preventing critical transitions. Next, I describe Niklas Luhmann’s theory of self-referential social systems giving particular attention to the distinction between system and environment, the communicative construction of the environment from functionally differentiated social systems, and the role of self-descriptions. Then, I discuss how social systems theory may provide new insights to the analysis of critical transitions considering the performative effects of social communication, and how critical transitions theory represents a compatible alternative for a theory of social systems crises. Finally, I draw the conclusions from my analysis.

## 2 Marten Scheffer’s Theoretical Landscapes

The theory of critical transition has been developed in the last two decades in the field of complex systems research to address sudden transformations that ecosystems may undergo because of the loss of resilience. Internal behavioral lock-in mechanisms and pressures in the landscape accumulate until a threshold condition (or tipping point) is reached around which a strong response is triggered in system dynamics, thereby opening a phase of regime shifts and bistability (Scheffer et al., 2001; van Nes and Scheffer 2005; Scheffer and Westley 2007; Scheffer 2009; Scheffer et al., 2015; Dakos et al., 2019).

While the theory is aimed at explaining why a sudden catastrophic fold^1^ occurs in system dynamics, it is rather construed on a distinction between gradual systemic behavior and sudden shifts. Often ecosystem conditions such as climate, species diversity, nutrients, or toxic elements change gradually over time; in other occasions, the system does not even react to changes in the landscape until a threshold is reached. However, there are some situations in which the response of the system is folded backward, thereby triggering a sudden transition or regime shift.^2^ In these cases, the system presents alternative states or attractors between which it oscillates, shifting from regime *a* to *b* (Scheffer et al., 2001).

Usual explanations of this behavior resort to external factors or shocks. While this might be true for some rather obvious situations (hurricanes, species invasion, human-made interventions), in most of the cases the transition can be produced by relatively small system-internal fluctuations dynamically connected with landscape variations. Going back to the previous state is rather difficult because the system needs to undo the shifting conditions that the system itself has reinforced through positive feedback loops which amplify small initial changes to large ones (van Nes et al., 2016). Paradoxically, for having the possibility of shifting back from *b* to *a*, the system dynamics has to move before the tipping point that itself has produced. This resistance to moving back and remain in the contrasting regime is called hysteresis (Scheffer and Carpenter, 2003).

Thus, a critical transition and the subsequent regime shift are unusual yet rather common situations. It is unusual because catastrophic shifts require time to accumulate the effects of initial changes, amplify them through feedback loops, and reach the tipping point after which the system collapses into another basin of attraction; it is common because every system may undergo this trajectory and, when the initial conditions take place, the most probable outcome is precisely the critical transition to another regime. In ecosystem research, this dynamic history of the system can be understood under the concept of resilience (Holling, 1973; Scheffer et al., 2001; Scheffer and Carpenter, 2003).

There are different definitions of resilience, from the most popular to the more technical ones. An intuitive and widespread definition is “the capacity of a system to recover upon disturbance” (Scheffer, 2009, 101). Though simple, this conceptualization captures the historical movement of the system and its perturbations. Yet for a more precise, operational, and quantifiable definition, Scheffer (2009, 103) offers a sophisticated alternative: “(resilience is) the capacity of a system to absorb disturbance and reorganize while undergoing change so as to still retain essentially the same function, structure, identity, and feedbacks.” More recently, (Arani et al., 2021, 2) has defined resilience as the mean exit time, i.e., “the expected time it takes for the system to cross a threshold such as the border of an attraction basin.” Resilience, therefore, involves a dual aspect. On the one hand, it stresses the fundamental instability of systems while aiming to maintain stability and, on the other, it underlines the possibility of change within self-produced borders. To that extent, a critical transition is closely related to resilience not only in the sense that losing resilience may lead to a regime shift into another basin of attraction, but also in the rather paradoxical sense that demanding resilience from vulnerable social communities may lead to an accumulation of social distress, thereby reinforcing “the resilience of an already undesirable social-ecological state” (Stockholm Resilience Centre, 2016, 1; see also Costanza et al., 2007). To express this in a political language: weakness may lead to chaos, but too much control leads to revolution.

For a gradual, smooth walk through the ecological landscape, the system should avoid sticking to a behavior that affects its own resilience. This is difficult because adherence to behavioral patterns -or lock-in mechanism- is a common situation in nature and society that has evolutionary advantages (Scheffer and Westley, 2007). Lock-in mechanisms are operational feedback loops that gradually produce connections between otherwise loosely coupled elements, thereby forming a system network. They are crucial to differentiate functions and network domains at the organic level, to automatically recognize dangers and opportunities, to produce a reflexive self-consciousness at the level of individuals, to create identities in social groups and systems to which others and the system itself may continuously refer to recognize topologies and distribute resources. Lock-in mechanisms are thus a key behavior for producing identity and diversity in the social-natural world. However, the persistent self-reinforcing adherence to a behavioral pattern may also lead to blindness regarding the changing landscape conditions and to a severe mismatch between the performance of the system and the dynamic movements in the landscape. This causes inertia and loss of resilience (Scheffer, 2009). It is not easy to escape from a lock-in mechanism because they are a constitutive element of the system without which the system itself dissolves; however, remaining fixed to well-known parameters of behavior when the environment significantly changes, results in a consistent and cumulative loss of resilience until a minor perturbation triggers the critical transition to another regime.

Lock-in mechanisms exist not only in nature. With other names and models, they have been identified in different contexts. Decision theory refers to sunk-cost effects for situations when individuals or organizations persist in a model of action because of previously invested efforts, avoiding thus a proper assessment of the present (Arkes and Blumer, 1985). Animal research calls this the Concorde-effect, which is only identifiable in human animals (Arkes and Ayton, 1999). Organization theory recognizes another form of inertia called efficiency trap, namely, the insistence on exploiting a well-known niche instead of exploring new alternatives (Liu, 2006; Mouzas, 2006). In a similar way, resilience theory also refers to social-ecological traps, i.e., feedback interactions between human activities and ecosystem services and dynamics leading to vulnerable pathways and undesirable states (Janssen et al., 2003; Cinner, 2011; Stockholm Resilience Centre, 2016; Graziano et al., 2021). And the research on the collapse of ancient complex societies argues that many civilizational crises can be explained by the adherence to a mode of behavior that prevents society from innovation and adaptation to the changing conditions of its own social-natural landscapes (Yoffee, 2004; Schwartz and Nichols 2010; Tainter, 2017).

Considering the enormous theoretical relevance and practical consequences for the system’s behavior of both the loss of resilience and the lock-in mechanisms, a crucial question arises whether tipping points and critical transitions can be technically addressed in order to recognize the limits of social-ecological systems and strengthen their adaptive capacities under conditions of uncertainty. Accurate measures remain elusive because of mechanistic limitations (van Nes and Scheffer, 2007), yet there are some recurrent system behaviors that seem to precede tipping points and work as precursors or early warning signals of the approaching bifurcation. They are called DIORs, namely, dynamical indicators of resilience (van der Bolt et al., 2021) –two of the most interesting ones are critical slowing down and flickering. In the first case, the system presents a decrease in the rates of change and takes longer to recover from eventual perturbations. A high variance in the pattern of fluctuations is an indicator of this behavior which results from the fact that the basin of attraction is not sufficiently pronounced to concentrate dispersion. In the case of flickering, the system switches continuously back and forth between regimes *a* and *b* after a large perturbation. The switching variation increases before the threshold, thus producing a rather hectic state of bistability until the system collapses into one of the alternative regimes. Both types of early warning signals have been experimentally identified in fields such as ecosystems, climate transitions, plankton and bacterial populations, lake eutrophication, epilepsy, migraine strikes, depression, financial markets, and the collapse of ancient societies, among others (Scheffer and van Nes, 2006; Dakos et al., 2008; Scheffer, 2009; Scheffer 2010; Veraart et al., 2012; Wang et al., 2012; Scheffer et al., 2013; van de Leemput et al., 2014; Battiston et al., 2016; Carpenter et al., 2019; Rodríguez-Sánchez et al., 2020; Scheffer et al., 2021; van der Bolt et al., 2021).

The theory of critical transitions continues being developed by Scheffer and collaborators. A view on Niklas Luhmann’s theory of social systems can contribute to this development from a sociological point of view.

## 3 Niklas Luhmann’s Systemic Environments

Niklas Luhmann’s theory of self-referential social systems combines elements from different sources, disciplines, and traditions–certainly from the sociological and philosophical tradition, but also from mathematical logics, theoretical biology, second-order cybernetics, and complexity sciences. Among the theoretical offerings in contemporary sociology, systems theory enters the paradigm of complexity more explicitly and directly than others to provide an overarching theory of modern society (Luhmann, 2013). Considering language, architecture, and approach, it can perfectly dialogue with complexity theories in general, and with the theory of critical transitions in particular.

The theory began to be conceptualized in the mid-1960s as a further development of Talcott Parsons’ classical systems theory. While Parsons’ theory was based on the traditional paradigm whole/part, the concept of open systems, and the operation of interchange through input-output relations, Luhmann’s theory is based on the complexity paradigm, the concept of self-referential closed systems, and the operation of communication as a distinctive feature of social systems (Luhmann, 2006). One of the main contentions of Luhmann’s approach is that there is only one world society with internal differentiations evolutionarily produced by meaning-based self-referential autopoietic communication systems (Luhmann, 1990a, Luhmann, 2013). Individuals–deconstructed as a coupling of psychic and organic systems–are not a part of this society. They preserve their autonomy as bodies and consciousness in the environment of society, yet they are, structurally, strictly coupled to society through language and, therefore, contribute decisively to the operation of communication. To that extent, nature is conceived of dually: as an internal representation of society with different meanings depending on the position of the observer, and as a continuum of materiality supporting social communication (Luhmann, 1995).

Systems theory is composed of a well-integrated network of basic concepts -such as contingency, complexity, risk- and four sub-theories: communication, evolution, differentiation, and self-descriptions. They presuppose each other and are horizontally covered by the basic cross-cutting concepts (Luhmann, 2013). Far from attempting an exhaustive description of the theory, I concentrate on three elements that, in my view, may help for a dialogue with the theory of critical transitions: the distinction system and environment, the construction of the environment within differentiated systems, and the pragmatic force of self-descriptions.

Systems are not a well-demarcated container of components with clear borders whose elements and relations can be described in terms of a supermarket shopping list. Systems theory does not deal with pre-existing objects, but with the distinction between system and environment. The point of departure is not the unity but the difference. The world itself can be understood as a difference between actuality and potentiality in which actual states are not less real than the potential ones. The crossing (transition) from one state to another transforms potentiality into actuality and vice versa. The world is thus contingent, i.e., neither necessary nor impossible. It is characterized by an overabundance of possibilities that cannot be processed all at once. The world is thus complex, i.e., there are more elements and relations than the actualized ones (Luhmann, 1990a; Luhmann, 1990b). To deal with contingency and complexity, they must be reduced. Selectivity offers a mechanism for the reduction of complexity, namely, for finding a way through contingency and complexity. Selectivity means drawing a distinction in the world that recursively emerges as a system that, in turn, permanently draws a distinction with the environment through selectivity. Thus, reducing complexity is also a form of increasing complexity and producing self-diversity (Luhmann, 1990b). To cope with this, novel forms of selectivity are required that reintroduce the cycle complexity–reduction of complexity–production of complexity. System and environment are therefore co-original, and the boundary is always dynamic and under construction. They are not fixed entities that appear as self-evident for the observer. Rather, every system has to deal with its own difference and the self-produced differences of other systems.

Systems could be organic and natural, machines and technological structures, and there are also meaning-based systems such as the psychic and the social system. Social systems include different forms of interactions, loosely coupled groups, organizations, major functional systems such as science, economy, law, politics, among others, and the all-encompassing social system society, or world society (Luhmann, 1995). The operational mode of social systems is communication (Luhmann, 2013). As social systems recursively produce and reproduce their own differences through their own operations (e.g., payments in the economy, legal validity in law, collectively binding decisions in politics), every system is autonomous, yet they are intrinsically interdependent, e.g., the economy presupposes valid norms provided by law, law presupposes political power to enforce decisions, politics presupposes payments to implement political programs. Social systems are structurally coupled, therefore problems in one of them produce domino effects on the others, generalizing thus critical conditions all over the social system (Mascareño et al., 2016). But social systems are also coupled with both psychic systems, who introduce individual variations through language into the stabilized patterns of social communication, and with nature, which produces a wide range of perturbations in social communication. Nature, in turn, receives the material consequences of communication to which society gives different names: ecological dangers, resource depletion, contamination, climate change, or Anthropocene.

Because of the differentiation of society in multiple functional systems, each one with its own autonomous concerns and communicational patterns (Luhmann, 1997a), the perturbations from the natural environment are not univocally observed by social systems. While politics sees ecological dangers through public opinion, the economy observes resource depletion, tourism regrets the contamination of landscapes, science warns against climate change, media adopt the concept of the Anthropocene as an explanation of the current social-ecological crisis, and social movements combine most of these concepts to protest against society from within society. Society has no central bank for communications, so natural events have a distributed rather limited resonance in different social systems (Luhmann 1989). On the other hand, every system develops its own repertoire for responding to natural perturbations. This repertoire depends on the structural conditions of each system which are neither a primary nor even a secondary concern for other systems. While social movements demand the final closure of contaminating industries, the economy raises the question of production and employment; while science announces measures, standards and early warning signals to avoid the social-ecological collapse, international politics make concessions to present some success in the coordination efforts for a new environmental agenda; and while the media moralize about the melting of Antarctic ice, tourism organizes trips to enjoy the Antarctic ice for the last time. There is no doubt that society reacts to environmental problems, but this does not mean that these reactions can reduce or mitigate the environmental problems. As the resonance of natural events in communication is observed by systems with different rationalities, critical communication on critical events rather multiplies the sense of crisis with often contradictory or at least orthogonal views on the events.

Considering this, society cannot be conceived of as a hierarchy; it has no top and no center (Luhmann, 1984). Priorities change from day to day according to the resonance they find in the mass media, including social media. One day is global security after a terrorist attack and the next is a sexual scandal or a protest against the greed of Wall Street. Not that society cannot recognize social-ecological dangers. Deforestation, wildfires, endangered species, diversity loss, melting ice, climate change, massive red tides, pandemics have become part of the regular vocabulary of modern society. But precisely the multiplicity of references, the global character of problems, and the decomposition of society into contrasting systemic rationalities makes it difficult to design a common coordinated action. From the point of view of social systems theory, “social-ecological” means the paradox of an increased consciousness (resonance) of the interdependence, even the integration between nature and society, together with fragmentation in the modes of conceiving this relationship and dealing with its problems.

To cope with this indeterminacy communication produces self-descriptions: “Self-description means selection of distinctions and indications, of differences and identities; it means self-simplification as a requisite for complexity. Self-descriptions, then, have to be conceived as the necessity to produce contingent reductions. They can neither be avoided nor accomplished as a true picture of their object” (Luhmann, 1984, 66). “Climate change” is a self-description. This does not mean that climate change is an illusion, that there is no data and scientific evidence to support that the climate has changed, even dramatically in the last decades. However, the communicative formula “climate change” does not depend on the evidence, but on the reproduction of the multiple meanings contained in the self-description: a reference to scientific facts, a critique of the model of growth, a call for urgent action, a warning of an approaching social-ecological catastrophe. Self-descriptions do not grant direct access to the object, but they increase the possibilities of communicative success−i.e., of adopting the communication as a premise for ones’ own behavior (Luhmann, 1995)− by connecting different meanings under the name “climate change.” As Luhmann (2013, 179) argues: “(by means of self-descriptions) a system can surprise itself with itself and gain ever-new insights from itself.” This represents an important opportunity for the recognition of social-ecological problems: society loses accuracy in identifying and outlining these problems but gains in motivation to confront them.

## 4 Discussion: Communicating on Social-Ecological Transitions

Efforts of connecting theoretical approaches are not only an intellectual exercise. They are rather guided by a twofold goal: to illuminate some blind spots every theory has, on the one hand, and to contribute to increasing the correspondence between the complexity of the concrete problem and the complexity of the explanation, on the other.^3^ This is what I meant above by cross-fertilization. In my view, systems theory can contribute to the theory of critical transitions with a refined concept of social communication; and critical transitions theory can provide systems theory with an empirically informed and scientifically sound theory of complex systemic crises understood as critical transitions.

### 4.1 Semantics and Social Structures: Narrative of Progress Versus the Narrative of the Biosphere

Critical transitions and resilience theory are aware of the importance of meaning-making when it comes to transforming towards sustainable futures. As Folke et al. (2021, 852) argue, meaning-making requires “promoting new narratives that resonate, inspire, and provide hope centered on a plurality of criteria of worth and social inclusion. Here, we are concerned with the challenge of motivating a collective recognition of our interdependence with the biosphere and economic and political action based on that recognition.” Likewise, Carpenter et al. (2019, 4) contend that “collective imaginaries,” i.e., “representations that draw their authority from an empirical foundation, significant experiences of a community, and nonrational roots, play essential roles in guiding human motivation and action.” There is a clear understanding in resilience theory of the performative effects social communication, social semantics, and self-descriptions may have on reframing social-ecological critical situations.

The problem is, however, that not only those who recognize “our interdependence with the biosphere” develop “collective imaginaries.” The nineteenth-century narrative of progress and the twentieth-century narrative of modernization are still “narratives that resonate” in different centers of Western and non-Western life (Mascareño, 2007). The radical separation between nature and society is a primary component of these narratives. Through it, most nation-states in world society have structurally organized their relationship with nature in terms of resource exploitation and justify their policies as a way of overcoming poverty through growth, i.e., they also refer to the semantics of social inclusion and well-being as a moral foundation for postponing the attention to ecological dangers and emphasizing economic growth and employment (Beckerman, 1992; de Bruyn, 2000). Put differently, prevailing narratives of progress and modernization are supported by pragmatically successful and historically construed social structures.

More pointedly, the narrative of progress and its variations are a two-centuries-old semantics maintained by a tightly coupled network of social structures (institutions, organizations, systems), while the biosphere narrative supported by a social-ecological point of view has become symbolically generalized in the last two or 3 decades, as we gain awareness of our global social-ecological interdependence. The social structure for this narrative is just emerging. For its reproduction and generalization, both global adaptive governance (Folke et al., 2005) and a network of non-national global institutions that think biospherically are required. From a system-theoretical point of view, the success of the biosphere narrative depends not only on collective recognition and community experiences, but on the provision of functional equivalents to the social structures that support the narratives of progress and modernization, namely, structures with increased capacities of oversight and supervision that override the nature/society divide as well as the immanent restrictions, and even neglect of differentiated social systems towards social-ecological problems (Luhmann, 1997a; Willke, 1997, Willke, 2007).

Meaning arises from a continuous interaction between experience and expectation (Luhmann, 1990a, Luhmann, 2014). However, the transformation of meaning into a generalized symbolic self-description of society must be supported by social structures that can either meet expectations or can be called upon for compensatory returns when expectations are disappointed. As Luhmann (2013) argues, all-encompassing self-descriptions of the world as a whole are still underdeveloped. Human rights and cosmopolitan citizenship are two of them, however, both have a rather anthropocentric orientation. And while the emerging semantics of the Anthropocene is aimed at raising awareness of the human effects on the environment, its focus on the *anthropos* still runs the risk of observing nature through its adaptation to the conditions of existence of the human species. The virtue of the semantics of the biosphere lies in its ability to reconstruct the nature-society nexus as a complex, fragile, and highly sensible interdependent network. The systemic challenge is thus to produce institutions that live up to these standards.

### 4.2 Locked-in Communication: From Redundancies to Repetitions

As the theory of critical transitions argues, not only ecosystems may fall into a self-reinforcing adherence to behavioral patterns that affect their adaptive capacities and resilience. Concepts in social sciences such as sunk cost effect, Concorde effect, social-ecological trap, or efficiency trap show that human and organizational behavior is also prone to go through inertial feedback loops that lead to critical transitions (Scheffer, 2009). The question is whether social communication, as understood by Niklas Luhmann, may also be subject to these reiterative patterns that decouple the social system from the changes in the environment, leading them thus to a collapse.

From a systems theory point of view, social communication is always a matter of connectivity. For constituting themselves as self-referential systems, social systems have to connect one event of communication with the next one. They do this by accepting or rejecting communication offers. In both cases, communication can continue either with another information on the topic or reflecting on the conditions of acceptance or rejection of the communication. Crucial is to produce differences and avoid repetitions. In any case, the connectivity of communication depends at some level on producing redundancies, namely, on the possibility of accessing the same information through different sources or accomplishing the same function by means of different mechanisms. In this way, the system gains independence from particular relations and reinforces the accessibility to relevant communications (Luhmann, 1995).

Problems arise, however, when communication begins to repeat some selections. I suggest that when communication transits from the production of redundancies to production of repetitions, we confront a communicative lock-in. The problem takes place at the level of systemic programs, i.e., in the way in which social systems translate their respective functional codes into concrete operations. Through programs, systems offer options of selectivity to communication (Luhmann, 1995). The more diverse, redundant, and contingent these options are, the wider is the range of structural couplings the system can establish with the environment, and the more adaptive is thus its relationship with it. However, when systemic programs tend to suppress selectivity options (e.g., polarization in politics, monopolies in the economy, fundamentalism in the religious system, dogmatization of science), the memory function loses diversity to reconstruct the past and the oscillation of communication becomes limited and deflated (Luhmann, 1997b; Luhmann, 2013), thereby affecting the dynamic stability of couplings and the adaptiveness of the system to the environment. In other words, it becomes blind to the changes in the environment.

When this happens, the production of contingent, redundant options of selectivity is reduced to repetitive communications. For example, the problem is not the existence of many political parties, but the fact that we have to see the same political faces and hear the same political discourses election after election; the problem is not the volatility of prices but the fixation of prices and the presence of monopolies in the economy; neither is the problem that the industry produces plenty of goods and services that we buy, but their overproduction when there is no actual demand or the overexploitation of natural resources until depletion. Situations such as the use of anachronic laws to decide on new events, the recycling of articles in the academia, plagiarism in the arts and sciences, and teachers that just repeat textbooks in classrooms present a similar inertial, locked-in communicative pattern. In cases like these, repetition produces losses in the value of information and a new communicative event has little new to offer for supporting and fostering the connectivity of the process, constraining thus the self-referential operations and the cognitive openness of communication to new environmental events. To that extent, innovation at the level of organizations (political parties, firms, industries, banks, courts, universities, schools) becomes crucial for increasing the options of selectivity (redundancies) and avoiding the inertial effect of repetitions. Otherwise, they could end up reinforcing the inertial dynamics of the lock-in and bringing the system closer to a sudden critical transition.

As seen, like ecosystems, communication can also be captured by the dynamics of the lock-in mechanism. The 2008 financial crisis has been interpreted this way by Teubner (2011, 4): “Independently of the addiction of individuals, communication concatenate so that they would become caught up in compulsive engagement in an activity, despite lasting self-destructive consequences.” Haldane and May (2011) have argued in a similar fashion: if there is a premium to trading, banks continue supplying new instruments in financial communication. In my own social-ecological research on Chiloé Island, Chile, communicational controversies can be identified that open a clear view into communicative locks (Mascareño et al., 2018). After a massive dumping of dead fish into the sea by the regional salmon industry, a major red tide crisis affected the island in March 2016. A commission of scientists appointed by the government, after analyzing data of wind patterns, temperature of water, and marine currents, established that the dumping of dead fish did not correlate with the red tide outbreak, because the circulation transported the dumped material to the west and northwest. On the other hand, scientists of the international NGO Green Peace, based on similar data and satellite images, established that the circulation of waters led dead salmon to the southeast. So, in communication, 5,000 tones of dead salmon dumped into de sea swam in opposite directions. Facts and meanings were aligned in opposite ways; they became communicative locks from which actors could not escape. The controversy escalated to massive and violent protests of islanders who took over the island closing its accesses over a month, thereby producing a critical scarcity of food and supplies and posing a major political problem to the central government.

Communicative locks can severely reinforce social-ecological crises. In these cases, communication tends to repeat the past adding no informative value to new communications. This is particularly true in regional settings with a history of conflicts between communities, political authorities, and some type of extractive industry. Theoretically, self-referential social systems process every present event through a distinction between memory and oscillation. While memory brings about a selective reconstruction of the past in communication, the oscillator function opens the system to possible future selections that, in turn, are connected to memory (Luhmann, 1997b). This means that a history of conflicts between different agents sharing a social-ecological landscape will most probably align new facts with a future state of the conflict–as in the form of a self-fulfilling prophecy. As every complex system, communication is also sensitive to initial conditions: it preselects conflict (memory) and then chooses the type of conflict it has to deal with (oscillation). This reproduces a logic of failure and becomes a relevant communicative lock-in mechanism that may either accelerate or trigger social-ecological critical transitions as well as major political crises. Therefore, a theory of critical transitions may take this controversial, conflictive character of communication into consideration–it is based on reiterative patterns of communication that produce blindness to alternative ways of interpreting new events.

### 4.3 Communication: The Ultimate Oracle Machine for Early Warning Signals

In his studies on computability and non-computability, Türing (1939) expressed the idea of the oracle machine that is neither a component of a mechanical computer nor a human superintelligence but expresses an incomputable step that gives answers to unresolvable questions. In Turing’s words: “Let us suppose that we are supplied with some unspecified means of solving number-theoretic problems; a kind of oracle as it were. We shall not go any further into the nature of this oracle apart from saying that it cannot be a machine” (Türing, 1939, 172–173).

In systems theoretical terms, communication is a non-computable suprahuman emergent reality in which answers are given to even not yet formulated questions. It thus correlates with the knowledge and non-knowledge of the world in the form of actuality and possibility. In this sense, communication is not complete, it does not resolve Gödel’s problem of incompleteness (Gödel, 1931), but because of this is dynamic and poietic −autopoietic−, always connecting one event to the next one in an infinite number of topics and contributions through which individuals and systems exchange impressions about the world, share expectations and anticipate possible futures. Because of the emergent, autonomous character of communication, there always will be a discrepancy between the present future (the present image of the future construed in communication) and the future present (the actual present that will take place in the future) (Luhmann, 1995). The future is non-computable as such (Beckage et al., 2013). However, the oscillation between possible futures is always expressed in social communication. Only in this sense, communication works as an oracle machine in which early warning signals for social-ecological transitions can be identified and analyzed.

Hodge (2013), for example, has argued that in the communication of myths, science can discover references to facts that can anticipate future disasters. Three months before a volcanic eruption in south-east Mexico in 1982, Zoque people reported that the footsteps of Piowachue -a mythological female figure in Zoque culture- caused systematic tremors in the volcanic area that were ignored by the authorities. The eruption killed 2000 people and led to a massive migration from the area. Hodge (2013, 358) highlights that “the myth has practical value, encoding a system of early warning signs that were fatally ignored.” Local knowledge is the keyword here. More than scientists and political authorities, local observers develop a highly sensitive intimacy with the landscape they inhabit. They perceive small variations in ecosystem conditions that can anticipate major social-ecological transitions. Several empirical analyses confirm that community observers can act as an intelligent network of environmental monitoring, i.e., a community-based early warning system (e.g., Hall, 2007; Santha et al., 2014; Alessa et al., 2016; Matti and Ögmundardóttir, 2021; Rana et al., 2021; Tabor and Holland, 2021).

The analysis of Twitter communication before, during, and after a social-ecological red tide crisis on Chiloé Island, Chile, in 2016 shows that the critical topics during and after the crisis were already crucial for local actors long before the events (Mascareño et al., 2020). As explained above, the red tide crisis correlated with the dumping of 5,000 metric tons of dead salmon into the sea that was allowed by the Chilean maritime authority. A few days after the dumping, the major red tide crisis ever known in the southern hemisphere took place. The conflict then focused on the responsibility of the regional salmon industry regarding the crisis and the supervising capacities of the state. Because of their closeness with the fishing activity, deficiencies in technical and legal regulations on industrial fishing and salmon aquaculture were already clear for artisanal fishermen years before the crisis. As Twitter communication shows, utterances of artisanal fishermen warned against the problems that weak aquaculture regulations could bring forth to the environment and employment in the Chiloé archipelago. At least since 2013, that is, three years in advance, they communicate on the topic, including several peaks on the issue each year. In other words, the answer to the question of what triggered the 2019 critical transition on Chiloé Island was contained in the communication of local artisanal fishermen long before the critical events. As in the case of the Zoque myth, the warning of local actors was ignored.

Besides the capacity of communication to anticipate possible futures, the behavior of the two main early warning signals identified by the theory of critical transitions–critical slowing down and flickering–is also reflected in communication. As said, critical slowing down is characterized by a high variance in the pattern of fluctuations after a perturbation of the system (Scheffer and Carpenter, 2003; Dakos et al., 2008; Scheffer, 2010). Communication can mirror this with a high dispersion in the number of critical topics in a given situation. Different theories and approaches contend that before revolutions, major events of civil unrest, and institutional breakdowns, protests not only proliferate in number but also in the variety of topics they cover (Yoffee, 2004; Sewell, 2005; Tainter, 2017). In the case of Chiloé Island, for example, inhabitants considered the 2016 red tide crisis as “another layer in a long history of disappointments” (Mascareño et al., 2018), which includes topics such as health care, education, connectivity, employment, decentralization, and a conflictive political relationship with the central state. The basin of attraction of social resilience was already weak in Chiloé Island even before the crises. The red tide crisis was the final perturbation that triggered the systemic collapse.

Flickering, on the other hand, means that the system switches back and forth between opposite regimes (Wang et al., 2012). In communication, polarization follows a similar pattern: the system has no middle ranges, it moves between extremes. Different political and institutional crises have been preceded by an increasing polarization in political communication that continues during the critical event (McCoy et al., 2018; Nguyen, 2018; Berman, 2019; Au et al., 2021). In environmental research, topics such as climate change or global warming are highly controversial in social media communication between believers and disbelievers or between different governments (Pearce et al., 2014; Dahal et al., 2019; Tyagi et al., 2020; Al-Rawi et al., 2021; Sonnett, 2021) −this can also be a source for identifying correlations between the patterns of flickering in communication and environmental data.

Social-ecological research has largely recognized the relevance of meaning-making in the interaction between communities and ecosystems (Crane, 2010; Carpenter et al., 2019; Folke et al., 2021; van der Leeuw and Folke, 2021). Communication, as understood by Niklas Luhmann’s theory of self-referential social systems, is the ultimate machine for meaning making and, therefore, a highly productive source for the identification of early warning signals of upcoming critical transitions. Particularly the intimacy of local actors with the moods of the ecosystems provides social communication with abundant information on how social-ecological crises emerge and unfold.

### 4.4 Reframing Crises as Critical Transitions in Systems Theory

When observed sociologically, critical transitions theory becomes a powerful analytical and technical approach for the enhancement of social systems theory. Its explanation on the dynamics and consequences of a critical transition is construed upon relevant theoretical principles akin to systems theory, namely, the concept of complex system, the instability as a source of relative stability, and most importantly the paradoxical insight that the self-reproduction of a system through feedback loops may also lead it to a collapse. In other words, systems, in general, are essentially unstable and potentially critical.

Nonetheless, there is no theory of social crises in systems theory. Instead, Luhmann offers a theory of evolution with the functions of variation, selection, and restabilization that accounts for change, differentiation, and diversity, as well as for the emergence and destruction of systems (Luhmann, 2013).^4^ The specific problem of sudden regime shifts, as described by the theory of critical transitions, is not addressed in Luhmann’s theory. For similar problems, social theory has regularly used the concept of crisis, historically conceived of as a crucial point, a bifurcation between illness and health, a judgment after which redemption grants eternal life, a transitional period leading to a decision (Koselleck and Richter, 2006). In this vein, Luhmann understands the concept of crisis as a negative self-description of modern society. Crisis is a concept that reflects the impossibility of society to give a coherent account of itself: “The encompassing system is too large and too complex to be immediately understandable. Its unity is not accessible, neither by experience nor by action” (Luhmann, 1984, 59). When society describes itself as crisis, it recognizes the gap between the world of interactions and the global functioning of society; it triggers an alarming function that moves into action. With this, Luhmann confronts sociological critical theories that are more interested in criticizing society rather than understanding its functioning. As an alternative, he offers a theory of evolution, a theory of differentiation, and a theory of self-descriptions through which we can recognize the production of negative and positive self-descriptions.

In any case, that Luhmann considers crisis as a self-description of society does not mean that crises do not have an empirical correlate. The conditions of autonomy and interdependence of functional systems are a source of crises: “As one of the consequences of functional differentiation we even have to expect more or less permanent crises in some of the subsystems […] [functional differentiation] includes an awareness, even a prediction of continuing crises, time pressure and the need for restructurations which cannot even claim to open the doors for a better future” (Luhmann, 1984, 64, 65). Crises are thus conceived as friction, tension, or conflict among systems, but not as a consequence of the system’s own behavior in which the same mechanism that reproduces the system is the mechanism that can lead it to a collapse. This is what systems theory can learn from the theory of critical transitions.

Paradoxically, this is something that systems theory already knows. To increase their connectivity, self-referential systems produce differences by applying their own operations to themselves, and in doing so, they also reproduce blind spots that affect their interdependencies and connectivity with other systems. This is literally the definition of a lock-in mechanism, as formulated by the theory of critical transitions. In the language of systems theory: the autopoiesis of communication that supports the system may, in turn, produce an irreflexive repetition of communicative selections that decouples the system from the environment. In those cases, the social system engages in an overproduction of previously successful selections without noticing that the environment has changed or demands something else–put in technical terms, memory suppresses oscillation and the past overwhelms present selectivity (Mascareño et al., 2016; Cordero et al., 2017; Mascareño, 2018). Examples of this are manifold: the perseverance on dogmatic opinions despite evidence to the contrary, the attachment to normalized semantics without structural correlate, the insistence on exploitative patterns until resource depletion, the adherence to laws that have been overcome by social reality, the persistence on political inattentiveness to the point of a total loss of public trust, the increasing moralization of democratic politics until maximum polarization and confrontation. These and others are common situations in the dynamics of communication that are self-produced by social systems. Certainly, they trigger domino effects that interrupt interdependencies, but they are an endogenous outcome of communicative feedback loops that, as in ecosystems, may cause sudden regime shifts.

A social systemic crisis is thus an implosion of communicative reflexivity. The system enters into a hypertrophic dynamics of pure self-reference without other-reference that drastically limits the options for selectivity, thereby announcing the proximity of a major regime shift. This is the moment in which social actors realize that they are playing a role in a tragedy from which they can only escape by crossing the line and unleashing the crisis: revolutions after decades of oppression, war after hopeless negotiations, massive emigration after a life of exclusion, divorces after years of suffering, bankruptcy after long periods of default, illiquidity after a mechanic overproduction of financial instruments. The crisis, therefore, controls the hypertrophy of the system. This is its function. It decomposes the repetitive communicative sequence (lock-in) that triggers the critical transition and recomposes the double operation of self-reference and other-reference by reestablishing the possibility of selection from a contingent complex world.

The theory of critical transitions provides systems theory with conceptual and technical tools for reframing crises in sociological tradition. Doing this does not require sociology to abandon conceptual formulas such as critique or self-description. Rather, it invites to rethink their position in an expanded theoretical architecture.

## 5 Conclusion

In this article, I have shown how Niklas Luhmann’s theory of social systems can contribute to Marten Scheffer’s critical transitions theory with a robust, complexity-based approach to social communication that can offer complementary forms of addressing social-ecological transitions. The performative effects of self-descriptions, the tendency of communication to engage in repetitive dynamics and feedback loops, and its capacity to provide both early warning signals of environmental problems and depictions of possible futures are crucial elements for refining our comprehension of social-ecological transitions. On the other hand, I also have shown that social systems theory can obtain from the theory of critical transitions a sophisticated concept of crisis as a critical transition that offers both technical tools for empirical examination and theoretical elements for further developing a general approach to change in complex systems. Several conclusions can be drawn from my analysis.

First, social-ecological transitions must not necessarily be critical. As Scheffer argues, ecosystems can also change gradually in time through a continuous adaptation to the landscape. However, this gradual adaptation may hide a dynamic process of accumulation of tensions that leads to sudden regime shifts and critical transition. These situations are never harmless to society. This is precisely what is contained in the adjective social-ecological. Social communication can ignore ecological problems, underrate them, it can even deny them by refusing the truth supported by the majority of scientific and historical evidence, but it can also act as a resonance box that amplifies the scale of the crisis by reproducing it in the multiple registers of communication–the particular consequences for different fields, organizations, communities, and individuals. All this is connected in communication and transforms different local experiences into one single experience of crisis with many faces. The communication of crisis can even connect present experiences with past experiences, reintroducing historical events into the present, and configuring the current event as a new layer of a cyclical social-ecological dynamics. The critical transition of the present becomes thus a long-term transition with several outbursts. This allows for the construction of stories, the identification of cumulative consequences, and the attribution of responsibilities. Communication is not a simple reflection of facts. Rather, it has its own complex dynamics and plays a crucial role in the production of social-ecological critical transitions.

Second, as every complex system, communication can also fall into repetitions and develop lock-in mechanisms that spread throughout the social-ecological networks reinforcing the dynamics of a critical transition. As I have argued, the inertial dynamics of social systems is characterized by an irreflexive repetition of memory that suppresses the oscillation of the system, thereby preventing it from contingent selectivity. In my view, stepping out from this spiraling effect requires an adaptive governance of memory, i.e., a reconstruction of the past that leads to a new deal in the present. A possible line of further research connecting resilience theory and social systems theory can be open here. The idea of an adaptive governance of memory follows the lines of the concept of adaptive governance proposed by Folke et al. (2005). But instead of dealing with the alignment of individuals, organizations, and institutions, it deals with the hypertrophic dynamics of communication when it turns into pure self-reference without other-reference. Memory distinguishes between remembering and forgetting to construe the narrative of the system. Forgotten elements are not lost in the void but remain as a possibility to reframe memory. Since communicative locks drastically reduce contingency to one repetitive choice, memory has no options to select, and the system reacts mechanically to the dynamic diversity of the present. Therefore, a governance of memory has to increase the available options to reestablish selectivity, i.e., the free oscillation of the system. It is thus directly aimed at decomposing communicative locks before they lead to the critical transition. Roundtables, negotiations, hybrid forums (Willke, 2007, Willke, 2014; Callon et al., 2009) combining different actors and views on a particular social-ecological problem are the mechanisms to increase the options of selectivity, disengage memory from its locks, and manage the transition before reaching the tipping point. Governance of memory is, therefore, a strategy to bring the system out of its inertia and move the actors to elaborate new options of coexistence with(in) the biosphere.

Third, diversity seems to play a relevant role here. In ecosystems research is usual to distinguish between diversity of species and functional diversity. Having regime shifts as a focal point, it is crucial for ecosystems to preserve functional diversity. The relevance of species diversity depends on the role each species plays in the niche: the less redundant the functions, the more relevant species diversity is (Scheffer, 2009). In social systems–and beyond moral considerations–diversity is crucial at many levels: diversity of individuals is a fundamental source of variation in social evolution, diversity of interactions is relevant for the experience of identity and difference, diversity of organizations is significant for functional redundancy, and diversity of functional systems is crucial for the success of differentiated communications. There are however trends in ecosystems evolution and social dynamics to reduce diversity to self-organized patterns of groups with similar species or individuals (Sakoda, 1971; Scheffer and van Nes, 2006). This increases the risk of behavioral and communicative locks that may lead to critical transitions. Social segregation, for example, depends on territorial, symbolic, and communicative locks that trigger polarization, conflict, and eventually major social critical transitions. As I have argued, in the diversity of communication we may find alternative forms of interpreting ecosystem events, we also find new options of selectivity when memory loses reflexivity, and we can even discover–particularly in local knowledge and communities–early warning signals that anticipate social-ecological problems. Hence, if not a value, social diversity is a relevant asset for coping with and managing social-ecological transitions.

Fourth, when it comes to social-ecological transitions and to matters that affect local or global biospheric conditions, theoretical reflections are not only an intellectual exercise but a performative act of intervention. They guide research and, on occasions, policy as well. Thus, meaning also belongs to the biosphere, it is present in every social-ecological event and immanently connected to actions. The cross-fertilization between the theory of critical transitions and the theory of self-referential social systems–besides mutually supplementing blind spots such as the complex dynamics of communication, on the one hand, and a theory of crisis as critical transition, on the other–contribute to enhance the correspondence between the phenomenon and our explanations. Improved forms of intervention can be obtained from this cross-fertilization. They may cover a wide range of options: from negotiations among local actors to decompose particular social-ecological locks, to probably one of the major communicative tasks of our times, namely, the construction and institutionalization of the narrative of the biosphere, a rather post-anthropocentric narrative of the nature-society nexus.

Finally, social-ecological transitions are an unresolvable mix of materiality and meaning. Unresolvable mix means that materiality is always provided with meaning and meaning turns regularly into materiality. Because of the classic divide between nature and society, we are used to think that nature is the domain of materiality and society the realm of meaning. However, when we think in post-anthropocentric terms, i.e., when we think biospherically, the boundaries become blurred. Even pure facts that claim not to be charged with meaning, have to mean it. And even the most abstract thought can be a premise for action–for mobilizing things in the world. Social-ecological systems are thus materially meaningful (or meaningfully material) systems that cannot be reduced to one of these sides. To that extent, a theoretical dialogue between the science of ecosystems and the science of society–as I have done in this article–is also a biospheric intervention. It contributes to overcoming the nature/society divide and invites to think whether the era of the biosphere can manage the effects of the so-called Anthropocene era. This would be probably the major social-ecological transition we have to expect in the future.

## Data Availability

The original contributions presented in the study are included in the article/supplementary material, further inquiries can be directed to the corresponding authors. And I did not detect any particular expressions.

## Footnotes

1 A conceptual idea recovered from René Thom’s catastrophe theory (Scheffer, 2009). As stated by Thom in one of the original formulations in Structural Stability and Morphogenesis: “Here we are most interested in morphogenesis, the process characterized by the formation and evolution of structures and of forms represented in *B*. These structures will be characterized by a given closed set *K* in *B x T* such that at each point of *K* the process changes its appearance. Such a closed set *K* will be called a set of catastrophe points of the process” (Thom, 1975, 18).

2 In the complex systems literature, these moments are called in different yet equivalent forms: bifurcation point (Sternberg, 2013), saddle-node bifurcation (Shivamoggi, 2014), critical threshold (Scheffer, 2009), catastrophic bifurcation (Kuznetsov, 1995), tipping point (Gladwell, 2002; van Nes et al., 2016).

3 The correspondence between phenomenon and explanations depends on how and to what extent the generative mechanism proposed in the explanation can reproduce, for independent observers, the actual operations involved in the actual event (Maturana, 1990). According to this, my hypothesis is that by considering different aspects of the operation of social communication, we can obtain a more complex view on the generative mechanism of social-ecological critical transitions, increasing thus the correspondence between phenomenon and explanation.

4 In this context, Niklas Luhmann uses René Thom’s concept of catastrophe for a particular situation in societal evolution, namely, when a new general principle of differentiation emerges: “We call the relatively rapid transition of a system to another principle of stability a catastrophe” (Luhmann, 2013, v2, 38). So far, this has happened only four times in societal evolution: from segmentary differentiation to center/periphery societies, then to stratification, and finally to functional differentiation. The emergence of new principles of differentiation in societal evolution does not abolish former principles. Rather, they become combined into complex arrangements under the primacy of the new principle. For example, modern society is primarily organized as a functionally differentiated society, yet inequalities account for stratification, urban settlements and hinterland for center/periphery differentiation, and nation-states, jurisdictions, or families for segmentary differentiation (Luhmann, 2013).
